# Confocal Laser Scanning Microscopy and Model Membranes to Study Translocation Mechanisms of Membrane Active Peptides

**DOI:** 10.3390/pharmaceutics14081699

**Published:** 2022-08-15

**Authors:** Corina Ciobanasu

**Affiliations:** Department of Exact and Natural Sciences, Institute of Interdisciplinary Research, Alexandru I. Cuza University, Boulevard Carol I, Nr.11, 700506 Iasi, Romania; vasilica.ciobanasu@uaic.ro

**Keywords:** cell penetrating peptides, antimicrobial peptides, homing peptides, TAT peptide, tLyP-1 peptide, NKCS peptide, confocal microscopy, peptide-membrane mechanism, internalization peptide kinetics

## Abstract

Membrane active peptides hold great potential for targeted drug delivery systems and understanding their mechanism of uptake is a key step in the development of peptide based therapeutics and clinical use. Giant unilamellar vesicles are cell-sized model membranes that can be individually observed under the microscope. The lipid composition of these membranes can be controlled, and interaction with peptides and changes induced by the peptides can be directly followed. Relevant information on the specific steps of peptides uptake can be obtained using membranes of different lipid composition. The present work provides a selection of dynamics and kinetics of peptides at interaction with model membranes of different lipid composition. The systematic peptide-membrane interaction was investigated by laser scanning confocal microscopy. The peptides used in this study neither internalized nor induced pore formation in neutral membranes composed of phosphatidylcholine and cholesterol. In membranes with anionic phosphatidylserine or cone-shaped phosphatidylethanolamine, all peptides internalized but only two of them were able to form pores, showing that the length of the peptide, the numbers of the arginine amino acid or the length of the α–helix are also relevant for the penetration efficiency of peptides.

## 1. Introduction

In the past decade, selective and effective drug delivery with peptides has attracted much attention as systems intended to minimize side effects on normal cells and a controlled distribution of toxic drugs to pathologic cells. Successful administration of a drug or a macromolecule comprise a few steps such as long circulation, penetration of the membrane, internalization in cells and endosomal release after endocytosis. Classical strategies for drug delivery like liposomes, viral vectors, microinjection or electroporation have poor specificity, immunogenicity, reduced efficiency and undesired high toxicity [1]. Cell penetrating peptides (CPPs) are short amino acid molecules with the remarkable property to surpass the plasma membrane barrier and reach the cellular interior. Moreover, such molecules can be used to deliver a plethora of cargoes inside cells, varying from fluorophores or magnetic nanoparticles to drugs, DNA or even liposomes [2]. In contrast to the aforementioned delivery methods, penetrating peptides can cross the plasma membrane in a noninvasive manner, and have low toxicity and high efficiency. To date, CPPs have been successfully used as tools in the diagnosis and treatment of different diseases, and some of them have entered different phases of clinical trials [3].

Additionally, some peptides, like antimicrobial peptides or a few homing peptides, act as drugs themselves. Antimicrobial peptides (AMPs) are molecules that exist in different organisms including humans as part of their innate immune system and have the ability to destroy bacteria, fungi or viruses. AMPs, as natural molecules, represent a good alternative to conventional antibiotics in the context of antibiotic resistance issues. More than 3000 antimicrobial peptides have been discovered so far and more than 200 were approved by the FDA, and the US Food and Drug Administration [4]. Homing penetrating peptides (HPPs) have the special property of specificity for receptors of normal cells or cells in pathological conditions, especially cancer. These HPPs were identified by in vivo phage display technology and were successfully used for delivery of therapeutic or imaging agents after clinical trials [5]. Understanding the mechanisms by which these peptides are internalized into the cells and then released from the endosomes are very important steps in their process of development as therapeutic or diagnostic systems. The process of translocation also depends on physical-chemical factors of peptides and membranes like amino acid composition, structure, concentration, lipid structure, curvature of the membrane, structure of the cargo, etc. Considering all of these, the energy dependent mechanisms like endocytosis and energy independence are used by the peptides for internalization [6]. The passive mechanisms include direct membrane translocation, the formation of inverted micelles or through pore formation and carpet mechanism [7]. Despite several studies on their activities and applications in drug or diagnostic tool delivery, the mechanisms of internalization of these peptides have not been entirely deciphered.

A very convenient and accurate system to study the energy- independent mechanism of internalization is represented by lipid model membranes, which are giant unilamellar vesicles (GUVs). The major advantage of model membranes is the possibility to control and modify the chemical composition and physical properties systematically. GUVs are comparable in size and curvature of the cells. The interaction of peptides with the membranes is the very first step of internalization and model membranes enable the understanding and deciphering of various factors contributing to this process. Moreover, GUVs can be easily analyzed by fluorescence microscopy due to their micrometer sizes.

Fluorescence microscopy is by far the most common technique utilized to track different events or molecules in cells. An area of important progress in this field is represented by confocal laser scanning microscopy, which makes possible the non-invasive in vivo imaging and tracking of fast processes, molecules and molecular interactions in cells with high spatial and time resolution [8]. In addition, by sectioning the sample along the z axis, 3D images can be obtained with high precision by using this technique. Using different combinations of fluorescent labels and different color channels simultaneously, confocal microscopy makes following the behavior of peptides and changes in the lipid membrane in real time possible, even at the single molecule level [2]. Fluorescence microscopy contributed significantly to efforts to understand how peptides translocate the membrane and exert their biological activity. Different mechanistic aspects can be revealed by the simultaneous observation of peptides and membranes through colocalization of peptides with different lipids or intracellular targets, in a single experiment. Moreover, the possibility of fluorescence quantifications provides the opportunity to precisely evaluate the interaction of peptides with the membrane in spite of light microscopy limitations.

In this focused work, I have attempted to provide a selection of the range of dynamics and kinetics of penetrating peptides when interacting with model membranes of different lipid compositions and properties investigated by laser scanning confocal microscopy. For that, four types of peptides were chosen, with representative different structures and properties, and their accumulation and internalization was tested on GUVs with peculiar lipid compositions and features.

TAT peptides are the very first discovered cell penetrating peptide and by far the most studied peptide as a delivery tool. We previously showed that TAT peptide is able to form nanosizied pores in giant unilamellar vesicles as passive mechanisms of membrane translocation [9]. Then, NKCS is an antimicrobial peptide derived from NK-2 peptide, a natural occurring molecule isolated from porcine small intestine. NKCS has a cysteine replaced by serine compared to the parent NK-2 at position 7 for better stability. This peptide enters the GUVs mimicking the cytoplasmic membrane of bacteria by a pore mechanism as well [10]. NKCS-[15-27] is a derivative of NKCS, corresponding to the region of C-terminal fragment from residue 15 to 27. tLyP-1 is a tumor homing peptide, the truncated linear form of cyclic peptide LyP-1. tLyP binds to the neuropilin (NRP1 and/or NRP2) receptor overexpressed in glioma, breast cancer, prostate cancer and tumor vasculature [10,11].

## 2. Materials and Methods

### 2.1. Peptides

TAMRA-TAT (TAMRA-YGRKKRRQRRR) peptide was purchased from Eurogentec, Belgium. The TAMRA-TAT peptide has molecular mass of 1972.3 g/mol, a net charge of +8 at pH 7.0 and an isoelectric point 12.41.

NKCS, NKCS-[15-27] and tLyP-1 peptides labeled with the fluorescent molecule Dy647P1 (Dynomics) were synthesized by Biosyntan (Berlin, Germany) in purity >95%. The fluorescent dye was coupled to the C-terminus through an additional cysteine as maleimide. The peptide were synthesized using the Fmoc/But strategy [12] on SYRO instrument, MultiSynTech, Germany. Peptide purity was verified by the manufacturer by LCMS 2020 and analytical HPLC (Shimadzu, Kyoto, Japan). The peptides were synthesized with an amidated C-terminus. Prior to use, the lyophilized peptide was dissolved in double distilled water at a concentration of 1 mM and then preserved at −20 °C between the experiments.

Dy647P1-NKCS (Dy647P1-KILRGVSKKIMRTFLRRISKDILTGKK) peptide has Mw of 4005 g/mol, a net charge of +10 at pH 7.0 and the isoelectric point 12.98.

The Dy647P1-NKCS-[15-27] (Dy647P1-LRRISKDILTGKK) peptide is the second helix of NKCS, with Mw 2346 g/mol, net charge +5 at pH 7.0 and an isoelectric point of 12.54.

Dy647P1-tLyP-1 (Dy647P1-CGNKRTR), with Mw 1642 g/mol, has a net charge at pH 7.0 of +3 and isoelectric point of 12.50.

**Figure 1:**
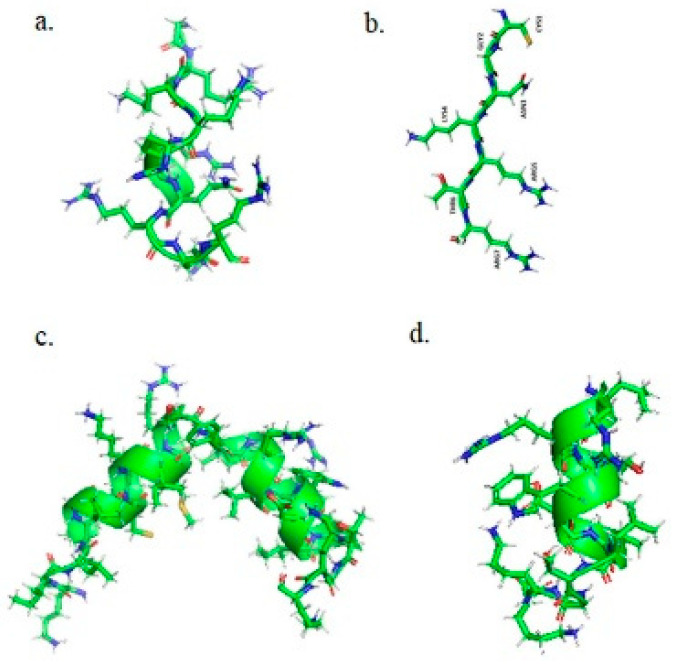
Secondary structure of the peptides used in this study, generated with pyMol 2.1. (**a**) TAT peptide, (**b**) tLyP-1 peptide, (**c**) NKCS peptide, (**d**) NKCS-[15-27] peptide.

The structures of peptides generated with pyMol 2.1 are presented in Figure 1.

### 2.2. Lipids, Fluorescent Tracers and Reagents

The phospholipids POPC (1-palmitoyl-2-oleoyl-*sn*-glycero-3-phosphocholine), POPE (1-palmitoyl-2-oleoyl-*sn*-glycero-3-phosphoethanolamine), and POPS (1-palmitoyl-2-oleoyl-1,2-*sn*-glycero-3-phosphoserine), were delivered by Avanti Polar Lipids (Alabaster, AL, USA). AlexaFluor^TM^ 488 C_5_ Maleimide was purchased from Thermo Fischer Scientific (Karlsruhe, Germany). Cholesterol (chol), sucrose, glucose and HEPES ((4-(2-hydroxyethyl)-1-piperazineethanesulfonic acid) were obtained from Sigma-Aldrich/Merck (Schnelldorf, Germany).

### 2.3. Preparation and Observation of Giant Unilamellar Vesicles (GUVs)

Giant unilamellar vesicles (GUVs) were obtained by the electroformation method as previously indicated [13]. For that, 30 µL of different lipid mixtures (detailed in the Results section) were dissolved in chloroform and spread on indium tin oxide (ITO) glass slides (Sigma-Aldrich, Schnelldorf, Germany). After drying, the lipid film was gently hydrated with 200 mM sucrose dissolved in double distilled water, mixed with a fluorescent tracer, Alexa Fluor 488-maleimide (AF 488), at a concentration of 10 µM. The freshly prepared GUVs were then transferred in 180 mM glucose solution containing 20 mM Hepes, with a pH of 7.5. The samples were observed by confocal microscope at room temperature.

### 2.4. Confocal Laser Scanning Microscopy (CLSM) and Quantitative Fluorescence Measurements

The experiments were performed using a confocal laser scanning microscope (Nikon Ti-E, Tokyo, Japan), equipped with LU4 Four-Laser Module with AOTF, a Plan Fluor 40x DIC M N2 objective and a DS-F1camera. An Alexa Fluor 488 tracer was excited using a line of 457–514 nm argon ion laser, TAMRA-TAT peptide with a diode laser emitting at 561 nm, and Dy647P1-peptides with a 642 nm diode laser. To observe the accumulation and internalization of peptides and the efflux of fluorescent tracers, the freshly prepared GUVs were transferred to a solution of glucose containing 2 µM fluorescently labeled peptide, to provide a homogeneous distribution of peptides to membranes. For each type of experiment, at least 10–15 GUVs with a diameter of 30–40 μm from two independent experiments were analyzed. Successive images were collected using the Nis-Elements Advanced Research imaging software (Nikon) and then quantification of fluorescence was performed using Fiji ImageJ [14]. The resulting data were then analyzed with Origin Pro 8.5 (OriginLab Corporation, Northampton, MA, USA).

## 3. Results

This study investigates the internalization of some peptides with distinct structures and properties in model membranes of different lipid compositions with confocal laser scanning microscopy.

### 3.1. Penetrating Properties of Peptides in GUVs

In our previous work, we demonstrated that interaction of the peptides with the lipid membrane and the penetration ability depends strongly on lipid composition [2,13]. Confocal microscopy is a very convenient and accurate method to distinguish between effects of peptides with different structures on membrane lipids.

For the present study, GUVs mimicking the mammalian plasma membrane were prepared as follows: POPC/cholesterol 80/20 mol%, POPC/POPE/cholesterol 50/30/20 mol% and POPC/POPS/cholesterol 50/30/20 mol%. The peptides fluorescently labeled (see Figure 1 for structure) were added at a concentration of 2 µM and the experiments were performed at room temperature.

**Figure 2:**
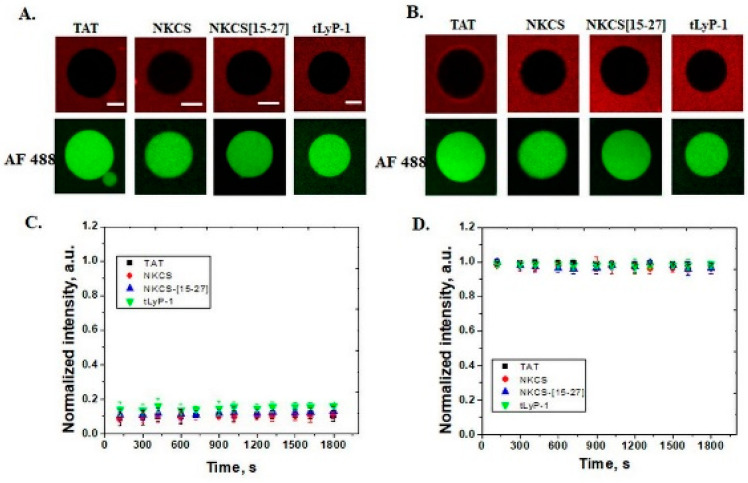
Internalization of peptides and efflux of Alexa Fluor 488 from POPC GUVs. (**A**) Interaction of peptides (TAT, NKCS, NKCS-[15-27] and tLyP-1) with GUVs containing POPC/chol 80/20 mol% after 5 min and (**B**) after 30 min. Neither peptide internalization nor AF 488 efflux can be observed even after 30 min as shown in the graphs (**C**), internalization of peptides (TAT (■), NKCS (●), NKCS-[15-27] (▲) and tLyP-1 (▼)) and (**D**), efflux of the fluorescent tracer. Error bars indicate the standard deviation. Scale bars correspond to 20 μm.

In neutral membranes comprised of POPC and cholesterol, peptides neither internalize nor induce the release of the internal fluorescent tracer Alexa Fluor 488, regardless of the structure, even after 30 min of incubation, as it can be observed in Figure 2.

**Figure 3:**
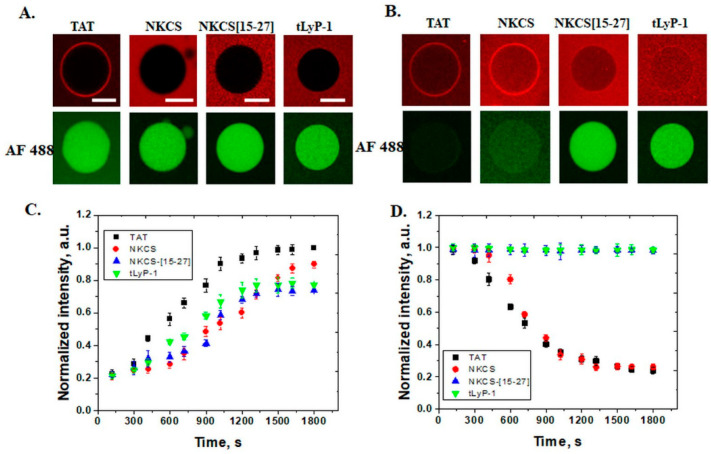
Internalization of peptides and efflux of Alexa Fluor 488 from POPE GUVs. Interaction of peptides (TAT, NKCS, NKCS-[15-27] and tLyP-1) with GUVs containing POPC/POPE/chol 50/30/20 mol% and release of AF 488, in the lower panel, (**A**) after 5 min and (**B**) after 30 min. (**C**) The time dependence of the normalized fluorescence intensity measured inside GUVs, for internalization of the four peptides (TAT (■), NKCS (●), NKCS-[15-27] (▲) and tLyP-1 (▼)) and (**D**) efflux of AF 488 tracer. Error bars indicate the standard deviation. Scale bars correspond to 15 μm.

In membranes with of 30% POPE, all peptides were translocated and were observed inside GUVs after 30 min. Also, the peptides accumulated on the membranes, with a slightly higher intensity for TAT and NKCS peptides. The lytic effect was observed by the efflux of AF 488 for TAT and NKCS, indicating the pore mechanism of internalization for these peptides (see Figure 3). The NKCS-[15-27] and tLyP-1 peptides internalized without the release of AF 488, suggesting a direct translocation mechanism.

**Figure 4:**
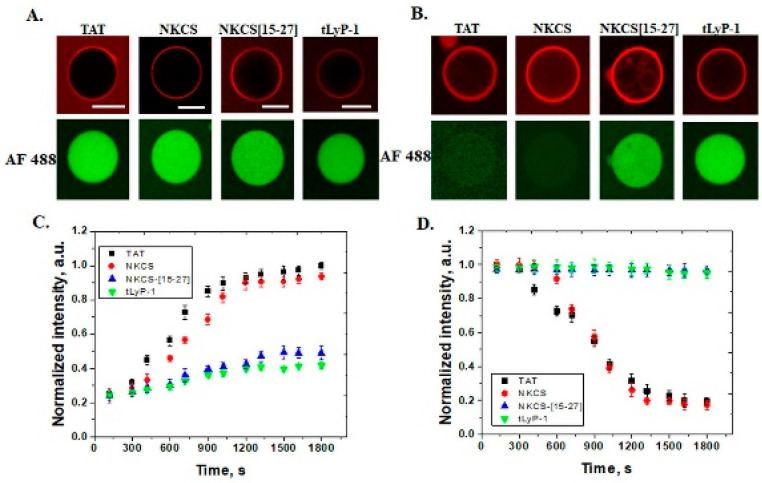
Internalization of peptides and efflux of Alexa Fluor 488 from POPS GUVs. Interaction of peptides (TAT, NKCS, NKCS-[15-27] and tLyP-1) with GUVs containing POPC/POPS/chol 50/30/20 mol% and release of AF 488 in the lower panel, (**A**) after 5 min and (**B**) after 30 min. (**C**) The time dependence of the normalized fluorescence intensity measured inside GUVs for internalization of the four peptides (TAT (■), NKCS (●), NKCS-[15-27] (▲) and tLyP-1 (▼)) and (**D**) efflux of AF 488 tracer. Error bars indicate the standard deviation. Scale bars correspond to 20 μm.

In anionic membranes, with 30 mol% POPS, a similar effect to POPE was observed (Figure 4). TAT, NKCS, NKCS-[15-27] and tLyP-1 peptides were internalized in these GUVs, but only the first two were able to form pores. In this case, a very strong accumulation of peptides on membranes was observed. Additional to the values of intensity measured inside GUVs, the internalization of peptides can be demonstrated by their accumulation in internal membranes of multivesicular GUVs, as can be observed for NKCS-[15-27] in Figure 4B. The same multivesicular GUVs indicated no lytic effect induced by the peptide, as well.

Confocal microscopy can reveal information about the pore formation mechanism but also about the size of the pores. For that, molecules with different sizes, like dextrans, can be encapsulated in GUVs and, depending on the released molecule, the diameter of the pore can be estimated. We previously showed that fluorescently labeled dextrans of 3 kDa were released from anionic GUVs but not from PE containing GUVs, in the presence of TAT peptide, indicating pores up to 1.3 nm in diameter [9].

### 3.2. Kinetics of Peptide Internalization

With a confocal microscope we can collect images showing the accumulation and internalization of peptides, but these images can be used for quantification of the observed effect by measuring the mean fluorescence intensity in a region of interest, GUVs number or the percentage of liposomes.

For kinetic traces, the time course of fluorescence intensity corresponding to peptides and Alexa Fluor 488 inside GUVs was measured. The data from three experiments were averaged and then fitted with a single rising exponential function to determine the values of internalization rates:

F(t) = 1 − exp (−k_int_ × t) (1)

where t is time and k_int_ is the peptide internalization rate constant.

The intensity corresponding to release of tracer Alexa Fluor 488 from GUVs in the presence of peptides was also measured to determine the efflux rates, using a single exponential decay function:

F(t) = exp (−k_eff_ × t) (2)

where t is time and k_eff_ is the peptide efflux rate constant.

The values of the internalization rate for membranes containing anionic lipids POPS were higher than POPE membranes (See Table 1).

### 3.3. Effect of Lipids on Activity of Peptides

We previously observed that the activity of peptides depends not only on their structure but on the lipid composition of membranes as well. While the TAT or NKCS peptide showed no lytic activity on membranes composed of zwitterionic phosphatidylcholine (PC), the situation changed when phosphatidylethanolamine (PE) and anionic phosphatidylserine (PS) when they were added to the composition of the membranes [9,10]. Thus, TAT peptides started to internalize and form pores at 30 mol% PS or 20 mol% PE.

Next to phosphatidylcholine, phosphoethanolamine and the anionic lipids, an important role in the composition of plasma membranes of mammalian cells is represented by cholesterol. Chol is a steroid lipid and is crucial in maintaining membrane fluidity. Like the phospholipids previously mentioned, cholesterol is amphipathic and orients with the polar headgroup to the aqueous medium. Cholesterol reduces membrane fluidity by forming strong interactions with phospholipids through its rigid steroid ring structure [15].

**Figure 5:**
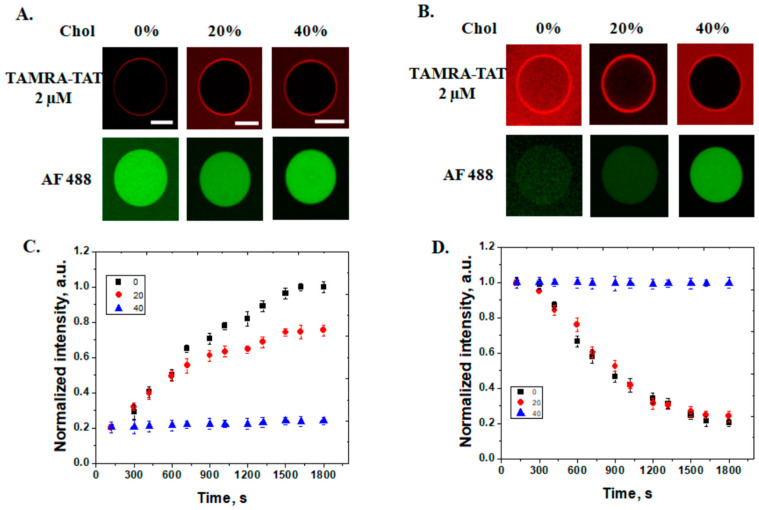
Internalization of TAMRA-TAT peptides and efflux of Alexa Fluor 488 from cholesterol containing GUVs. Interaction of TAT peptides with GUVs containing POPC/POPE/POPS/chol 70/20/10/0 mol%, POPC/POPE/POPS/chol 50/20/10/20 mol%, and POPC/POPE/POPS/chol 30/20/10/40 mol% and release of AF 488, in the lower panel, (**A**) after 5 min and (**B**) after 30 min. (**C**) The time dependence of the normalized fluorescence intensity measured inside GUVs, for different concentrations of cholesterol (POPC/POPE/POPS/chol 70/20/10/0 mol% (■), POPC/POPE/POPS/chol 50/20/10/20 mol% (●), and POPC/POPE/POPS/chol 30/20/10/40 mol% (▲)) and (**D**) Efflux of AF 488 tracer. Error bars indicate the standard deviation. Scale bars correspond to 20 μm.

In this study, the concentration of cholesterol was gradually increased in GUVs composed of POPC, POPE and POPS from 0 to 20 and 40 mol%. Indeed, while the proportion of cholesterol increases, the permeability of membrane for TAT peptide decreases, and neither the internalization of the peptide nor the efflux of the fluorescent tracer could be registered at 40 mol% chol (see Figure 5). Measured kinetics showed values of (0.77 ± 0.13) ×10^−3^ s^−1^ for GUVs without chol and (1.3 ± 0.1) × 10^−3^ s^−1^ for 20% chol for internalization of peptides and (1.49 ± 0.32) × 10^−3^ s^−1^ for 0 chol and (1.2 ± 0.1) × 10^−3^ s^−1^ for efflux of AF 488.

## 4. Discussion

The delivery of biologically active molecules and imaging agents with peptides is a dynamic and continuously developing field. Thus, many efforts have been made in understanding their cell internalization mechanisms, properties, ways to conjugate them to obtain new biologically active compounds or in the area of developing new methods to decipher the activities of peptides. These methods include flow cytometry, confocal microscopy, fluorimetry, circular dichroism, radioactivity, nuclear magnetic resonance, X-ray crystallography and mass spectrometry [16,17].

In this work, the focus is on a selection of peptides from three main classes, having different structures and properties: cell penetrating, antimicrobial, and homing peptides. These peptides are investigated by confocal microscopy, in relation to lipid membranes. As we previously observed, the activity of peptides was determined by the lipid composition of membranes. While all peptides do not internalize in neutral GUVs composed of PC and chol, in anionic or PE containing liposomes, peptides behave differently depending on the structure and amino acid composition. POPE is a neutral phospholipid in the shape of a cone and it is prone to form inverted micelles. It is the second most abundant lipid plasma membrane in eukaryotic cells [18]. In prokaryotic cells, PE is the most encountered, representing more than 70% of membrane lipids in *Escherichia coli* [19]. Phosphatidylserine (PS) is the most common negatively charged phospholipid in eukaryotic plasma membranes. In normal cells, it is found in the inner leaflet of the membrane and in endosome membranes [18]. In cancer cells, PS is overexpressed in the external leaflet of the plasma membrane and for this reason can be used as a target for cancer therapy [20].

In GUVs that contain 30 mol% PE, all peptides internalize, but only TAT and NKCS are able to form transmembrane pores through which the fluorescent dyes Alexa Fluor 488 can pass. A similar effect is observed for anionic GUVs with 30 mol% PS and shorter peptides, as NKCS-[15-27] and tLyP-1 peptides just translocate without generating pores. The role of lipid composition in model membranes for the internalization of cell penetrating peptides was also observed for nona-arginine (R9), a peptide that is able to form pores in DOPS and/or DOPE containing GUVs [21].

If we analyze the amino acid composition and structure, we can observe that both peptides are highly cationic, +8 and +10, respectively, and that the TAT is linear and NKCS is α–helix. NKCS-[15-27] is half of the NKCS helix with a charge +5 and tLyP-1 is linear with a charge of +3. Furthermore, if we look at kinetics, we can observe that NKCS is slower than the TAT peptide in internalization, while the AF 488 efflux is almost the same. These results lead to the conclusion that the number of basic amino acids is important for penetration activity. Some systematic studies have suggested a minimum of six or eight positive charges or arginines for peptide internalization [22,23]. Although it does not form pores, tLyP-1 can be observed inside GUVs, indicating a direct translocation mechanism. Furthermore, the length of α–helix for structured peptide is relevant for the penetration and pore formation ability since NKCS is internalized and forms pores while the efflux of AF 488 cannot be observed for NKCS-[15-27]. Obviously, the lytic activity of antimicrobial peptides is a complex process and depends on various physicochemical factors including structure, amino acid length, charge, hydrophobicity, amphipathicity, and self-association [24] and not least, on the lipid membrane. Shai et al. concluded that AMPs need at least 22 amino acids in order to be internalized [25], this number balancing the residues required for a turn and the number of turns necessary for formation of amphipathic faces. It has been suggested that peptide helicity is more relevant for toxicity (in neutral GUVs), while the positive charge is more important for antimicrobial activity (anionic GUVs). Reducing the net charge of an antimicrobial peptide to +4, both the hemolytic and antimicrobial effects were abolished. Increasing the charge up to +9 improved the antimicrobial activity, with low hemolytic effect. A further enhancement of the net charge over +10 resulted in a better antimicrobial response but with a dramatic increase in hemolytic activity [26]. In another study, Huang et al. chemically tuned the helicity of an AMP by replacing the original L-amino acids with D-amino acids on the non-polar/polar face of the α–helix. Better antimicrobial activity and reduced normal cell toxicity was observed for the residues replaced on the non-polar face [27]. The results presented in this paper are in agreement with these observations.

The peptides used in this study have no lytic activity for neutral membranes, suggesting a low toxicity effect for plasma membranes of eukaryotic cells. In membranes with PE or PS lipids in composition, TAT and NKCS peptides can be observed inside GUVs but they form pores as well, showing that these vectors can be successfully used for the delivery of small molecules with a size comparable with the fluorescent tags.

Membrane fluidity is another parameter controlling the permeability of peptides that can be analyzed with confocal microscopy. In GUVs with different concentrations of cholesterol, internalization of the TAT peptide is observed up to 40 mol% chol when the membrane is no longer permeable for peptides.

To summarize, confocal microscopy is a very reliable method to study the activity of different classes of peptides. GUVs are simple model membranes, but their simplicity is particularly important because it is possible to control the lipid composition or the ions from the medium. Also, do to their size it is possible to observe directly the dynamic changes induced by peptides using a confocal microscope. Together with qualitative observation, quantitative analysis is possible using confocal microscopy and GUVs. Studies on kinetics reflecting the internalization of peptides are very important in understanding the multi-step mechanism of translocation. Unfortunately, only a few studies are focused on this aspect [28]. This paper provides a systematic study on kinetics of different classes of peptides on membranes with a specific lipid composition concerning the internalization of peptides and the ability to form pores in these membranes.

## Figures and Tables

**Table 1:** Internalization of peptides and efflux rates of AF 488 in POPC/POPE/chol and POPC/POPS/chol containing GUVs.

Membrane	POPC/POPE/chol		POPC/POPS/chol	
**Peptide**	K_int_,s^−1^	K_eff_,s^−1^	K_int_,s^−1^	K_eff_,s^−1^
TAT	(1.13 ± 0.02) × 10^−3^	(1.25 ± 0.06) × 10^−3^	(1.32 ± 0.18) × 10^−3^	(0.90 ± 0.23) × 10^−3^
NKCS	(0.42 ± 0.24) × 10^−3^	(1.65 ± 0.14) × 10^−3^	(0.81 ± 0.13) × 10^−3^	(1.34 ± 0.06) × 10^−3^
NKCS-[15-27]	(0.20 ± 0.04) × 10^−3^	-	(0.50 ± 0.23) × 10^−3^	-
tLyP-1	(0.78 ± 0.03) × 10^−3^	-	(0.75 ± 0.27) × 10^−3^	-
